# The Effect of Periodontal Treatment on the Reactive Hyperemia Index. A 1-Year Follow-Up Pilot Study

**DOI:** 10.3389/fcvm.2022.851397

**Published:** 2022-04-14

**Authors:** H. C. M. Donders, E. O. Veth, M. A. Edens, A. W. J. van ’t Hof, J. de Lange, B. G. Loos

**Affiliations:** ^1^Department of Oral and Maxillofacial Surgery, Amsterdam UMC, Academic Centre of Dentistry Amsterdam (ACTA), University of Amsterdam, Amsterdam, Netherlands; ^2^Department of Oral and Maxillofacial Surgery, Isala Hospital, Zwolle, Netherlands; ^3^Department of Periodontology, Academic Centre of Dentistry Amsterdam (ACTA), University of Amsterdam and Vrije Universiteit Amsterdam, Amsterdam, Netherlands; ^4^Practice for Periodontology Zwolle (PPZ), Zwolle, Netherlands; ^5^Department of Epidemiology, Isala Hospital, Zwolle, Netherlands; ^6^Department of Cardiology, Maastricht University Medical Center and Cardiovascular Research Institute Maastricht (CARIM), Maastricht, Netherlands; ^7^Department of Cardiology, Zuyderland MC, Heerlen, Netherlands

**Keywords:** periodontitis, dental health, cardiovascular disease, endothelial dysfunction, EndoPAT—pulse wave velocity, periodontal treatment, cardiovascular prevention

## Abstract

**Background:**

Periodontitis is a chronic multifactorial inflammatory disease of the supportive tissues of the teeth. In more recent years, remarkable epidemiological and pathophysiological associations between periodontitis and cardiovascular disease (CVD) have been presented. Whether or not treatment of periodontitis is valuable for primary or secondary prevention of cardiovascular disease, has not yet been fully established. In this practice-based pilot study we focused on primary prevention of cardiovascular disease, by investigating the effect of periodontal treatment on the earliest detectable stage of CVD; endothelial dysfunction.

**Methods:**

Otherwise healthy periodontitis and non-periodontitis participants 45–70 years of age were included in the study. One year after completing periodontal (non-surgical and surgical) treatment of the periodontitis patients and 1 year after inclusion of the controls, all baseline measurements were repeated. Full-mouth examinations were performed by a periodontist to determine their Periodontal Inflamed Surface Area (PISA) score and other dental parameters. To assess the cardiovascular conditions, endothelial function through the reactive hyperemia index (RHI) assessed by the EndoPAT™, and several physical and biochemical parameters were measured.

**Results:**

21 patients with diagnosed, untreated periodontitis and 21 participants without periodontitis were included in this follow-up study. After periodontal therapy in the periodontitis patients, the PISA reduced significantly. The RHI did not show a significant improvement after treatment of the periodontitis patients (−0.1 ± 0.8, *p* = 0.524). Similarly, other secondary cardiovascular outcome measurements, hsCRP, total cholesterol, HDL cholesterol, triglycerides, HbA1c, and systolic blood pressure did not improve significantly after periodontal treatment. Controls did not show any significant changes in the RHI, in other CVD parameters and in the PISA after 1-year follow-up.

**Conclusion:**

In this practice-based pilot study, periodontal treatment did not improve the endothelial function in otherwise healthy adults with periodontitis. Future studies are needed to be of larger size and could focus on periodontitis patients with co-morbidities to investigate whether periodontal treatment has secondary preventive effect on endothelial function and other CVD parameters.

**Clinical Trial Registration:**

[www.ClinicalTrials.gov], identifier [ISRCTN55656827].

## Introduction

Periodontitis is a chronic multifactorial inflammatory disease of the supportive tissues of the teeth (1). It is the most common oral disease, and the sixth most common human disease, affecting 30–50% and approximately 10% of the global adult population in its most severe form (2, 3). Periodontitis starts with localized inflammation of the gingiva that is initiated by bacteria in the dental plaque. As a result of inflammation, the tissues surrounding the tooth are infiltrated by neutrophils, macrophages and activated lymphocytes. The subsequent state is periodontitis, with progressive loss of alveolar bone and tooth attachment and ultimately tooth loss. Bacteria and cytokines from the periodontal inflammatory lesions are dispersed throughout the body and acute phase reactants [C-reactive protein (CRP), fibrinogen] are produced (4). Although bacteria are necessary for periodontitis to take place, a susceptible host is also needed. Consequently, several risk factors for periodontitis have been established, including smoking, diabetes mellitus, socio-economic position, psychosocial factors, and genetic predispositions (5). Interestingly, some genetic risk variants for cardiovascular diseases show overlap with identified genetic variants of periodontitis (6).

The first study that found positive epidemiological evidence for the association between periodontitis and atherosclerosis was in 1989 by Mattila et al. (7). In more recent years, remarkable pathological and epidemiological associations between these two diseases have been presented, though without final conclusions (8–11). The explanation of the association between periodontitis and CVD generally fall into two categories: (a) microbial mechanisms, which through vascular invasion may locally affect the development of the atheroma lesions; and (b) inflammatory and immunologic mechanisms that directly influence the pathobiology of the atheroma lesion (12). Most pathophysiological links between these two diseases are based on the possible common inflammatory background between periodontitis and CVD (13, 14).

The possible link between CVD and periodontitis is of great public health importance because of the high prevalence of both diseases and the potential impact on public health if risk modification or therapeutic opportunities could be identified. Whether or not treatment of periodontitis is valuable for primary or secondary prevention of cardiovascular disease, have not yet been fully established. The majority of the intervention trials, aimed to study this purpose, has examined the effect of periodontal treatment on markers of systemic inflammation, focusing primarily on the role of common inflammatory pathways (15).

Endothelial dysfunction has been recognized as the critical junction between CVD risk factors and clinical disease, and is the earliest detectable stage of CVD (16). Tonetti et al. sought to assess the effect on intensive periodontal treatment on endothelial function measured by Flow-Mediated dilatation (FMD) of the brachial artery. The FMD was greater, and thus improved, in the intensive-treatment group than in the control-treatment group 60 days after therapy (absolute difference 0.9%; 95% CI, 0.1–1.7; *P* = 0.02) and 180 days after therapy (difference, 2.0%; 95% CI, 1.2–2.8; *P* < 0.001). The degree of improvement was associated with improvement in measures of periodontal disease (17). More recent studies did not find clinical evidence for a positive effect on endothelial function after periodontal therapy, and therefore a possible cause-to-effect relationship remains controversial (18, 19).

In this pilot study we focused on possible primary prevention of CVD in a practice-based setting. Accordingly, the primary aim of this study was to determine the effect of periodontal treatment on endothelial function as assessed by the reactive hyperemia index (RHI) in otherwise healthy patients with periodontitis. Secondary aims were to investigate the effect of periodontal treatment on other parameters, including high sensitive C-reactive Protein (hsCRP), (systolic) blood pressure, cholesterol (HDL, LDL, and total), triglycerides and glycosylated hemoglobin (HbA1c).

## Materials and Methods

### Study Design

This dental-practice based clinical intervention study was a 1-year follow-up, of our prospective cross-sectional study as published previously (20). The study was approved by the Medical Ethics Committee, Isala Academy, Zwolle, the Netherlands (NL43083.075.13) and has been registered in the ISRCTN trial registry with study ID ISRCTN55656827. All participants provided written consent for participation at baseline and at the time of follow-up. This study was performed in accordance with the Declaration of Helsinki guidelines for human research, 1964, and amended in 2013 (64th World Medical Association General Assembly, Fortaleza, Brazil). Data were collected, analyzed and interpreted by the authors.

### Participants

We included periodontitis patients and control subjects, between 45 and 69 years of age, without known systemic diseases and with at least 10 teeth. At baseline, all subjects were included in our previously published prospective cross-sectional study (20). The periodontitis patients received periodontal treatment after the baseline measurements. One year after completing periodontal treatment, these patients were recruited for participation in this follow-up study. The control group was also recruited for participation in this follow-up study, 1 year after the baseline measurements. All mentioned measurements below were performed at baseline and after 1 year of follow-up.

### Measures of Dental Health

All participants underwent a full-mouth periodontal examination performed by two trained periodontists at the Practice for Periodontology Zwolle (PPZ). Inter observer agreement of these two periodontists was examined by comparing individual measurements of 10 random participants. The statistically determined intraclass correlation coefficient of 0.81 represented an excellent reliability according to Fleiss (21). Periodontitis was initially diagnosed and staged according to the consensus report of the World Workshop on the classification of periodontal and peri-implant diseases and conditions (22). The Periodontal Inflamed Surface Area (PISA) score was applied. This scoring tool calculated the amount of inflamed periodontal tissue in square millimeters and quantified the total inflammatory burden resulting from periodontitis (23). The PISA score was calculated after extensive periodontal examination, including periodontal probing pocket depth (PD), plaque score and bleeding on probing (BOP). All measurements were performed on all teeth, on six sites per tooth using a manual periodontal standard probe. Rather than presenting mean pocket probing results and bleeding on probing scores, we regarded it essential for the current study to use the PISA score because it is the best integrative and overall score for quantifying the inflammatory burden posed by periodontitis. Moreover, it can be easily and broadly interpreted and applied by clinicians as well as patients.

### Measures of General Health

All participants completed questionnaires to collect data on their medical history, perceived health, parental history, lifestyle, socio-economic status, and oral hygiene.

At least 2 weeks after the periodontal examination, the participants were examined by a trained nurse at the Department of Cardiology of the Isala hospital, Zwolle. Physical examinations were performed, and blood pressure (BP), heart rate (HR), body mass index (BMI), waist-to-hip ratio (WHR), and electrocardiogram measurements (ECG) were obtained. Venous blood was collected to determine levels of high sensitive C-reactive Protein [hsCRP (mg/L)], total cholesterol (mmol/L), HDL-cholesterol (mmol/L), LDL-cholesterol (mmol/L), triglycerides (mmol/L), estimated Glomerular Filtration Rate (eGFR ml/min/1.73 m^2^) and glycosylated hemoglobin [HbA1c (%)].

### Measures of Cardiovascular Condition

As primary outcome we performed an endothelial function assessment by the EndoPAT™ (Itmar Medical, Israel), based on non-invasive Peripheral Arterial Tone (PAT) signal technology measuring endothelium-mediated changes in vascular tone using bio-sensors placed on the fingertips. This endothelial function assessment is validated vs. the invasive gold standard (intracoronary infusion of acetylcholine). It is a short and operator independent endothelial dysfunction test, that is easy practice or office based performable (24). The final result of the EndoPAT™ is the Reactive Hyperemia Index (RHI), which is a ratio of the post-to-pre occlusion PAT amplitude of the tested arm, divided by the post- to pre- occlusion ratio of the control arm. A RHI score of 1.67 and below correlates to endothelial dysfunction (25, 26).

### Periodontal Treatment

All periodontitis patients received periodontal treatment, including training in oral hygiene, and counseling on control of risk factors (e.g., smoking, alcohol usage and overweight/obesity). Supra- and subgingival bacterial plaque and calculus was removed by comprehensive, meticulous periodontal scaling and root planning performed by an experienced dental hygienist in the specialized periodontal practice using local anesthesia. When the non-surgical treatment had insufficient effect on the pocket depth and bleeding score, or when residual pockets deeper than 5 mm were still present, surgical treatment by a periodontist was incorporated. Surgical treatment consisted of procedures to reduce or eliminate periodontal pockets and create an acceptable gingival contour that facilitates oral hygiene and periodontal maintenance. Selective teeth that could not be saved were extracted. Finishing procedures included post-treatment evaluation with review and reinforcement of daily oral hygiene when appropriate. Thereafter, patients were enrolled in a periodontal maintenance program of 3–4 times a year performed by the dental hygienist of the specialized periodontal practice.

### Controls

The controls without periodontitis were educated in oral hygiene and were counseled on control of risk factors for periodontitis (e.g., stress and smoking). The dental health was maintained by their own dental hygienist 1–2 times a year.

### Statistics

Descriptive statistics [mean ± standard deviations (SD), Median (Q1–Q3) or numbers (%) of subjects] were used to present patient characteristics and results, depending on the distribution. Between group differences were tested by the Fisher’s exact test or Chi-square test for categorical variables and independent *t*-tests for quantitative variables. Change scores were calculated by subtracting old values from new values. Hence a negative value indicates a lower follow up value, i.e., decrease. Within group differences/changes were tested using the McNemar test for categorical variables and the paired *t*-test or the Wilcoxon signed ranks test for quantitative variables, depending on the distribution of change. The significance level was set at a *p*-value of 0.05. All analyses were performed using SPSS 26.0 (IBM Corp., Armonk, NY, United States). Figures were created using RStudio version 1.4.1717 (R version 4.0.3).

## Results

### Inclusion

At baseline, 27 patients with untreated periodontitis and 27 controls without periodontitis were included. Of these 54 participants, 12 subjects (6 periodontitis patients and 6 control subjects) were lost to follow-up, because of refusing to participate. Thus 21 periodontitis patients and 21 non-periodontitis controls were included in this follow-up study. There was no significant difference at baseline between the age, gender, BMI, PISA score, and endothelial function between the total 54 participants and the 42 participants included for follow-up.

### Participants

The study population consisted of 40.5% (*n* = 17) male and 59.5% (*n* = 25) female participants. The mean age was 54.1 ± 6.3 years. All participants were Caucasian. Fifty percent (*n* = 21) of these participants were tertiary educated, and 42.9% (*n* = 18) were secondary educated. Forty-one percent of the participants had a positive family history for CVD. The characteristics of these 42 participants are presented in Table 1. There was no significant change in periodontitis risk factors between baseline and follow-up (BMI *p* = 0.791, waist to hip ratio *p* = 0.259, smoking *p* = 1.000, alcohol intake *p* = 0.798).

**TABLE 1:** Characteristics of the study population.

	Total	Periodontitis	Control
	*n* = 42	*n* = 21	*n* = 21
Age	54.1 ± 6.3	55.4 ± 7.1	52.9 ± 5.4
**Gender**			
Male	17 (40.5)	10 (47.6)	7 (33.3)
Female	25 (59.5)	11 (52.4)	14 (66.7)
BMI	24.3 ± 3.2	25.4 ± 3.8	23.2 ± 1.8
Waist to hip ratio	0.9 (0.8–0.9)	0.9 (0.2–1.0)	0.9 (0.8–0.9)
Smoking	4 (9.5)	4 (19.0)	0 (0.0)
Alcohol servings/week*	4.0 ± 4.1	2.2 ± 2.7	5.4 ± 4.5
**Education**			
Primary	3 (7.1)	2 (9.5)	1 (4.8)
Secondary	18 (42.9)	8 (38.1)	10 (47.6)
Tertiary	21 (50.0)	11 (52.4)	10 (47.6)
Positive family history for CVD	17 (40.5)	7 (33.3)	10 (47.6)

*Alcohol servings/week: 8 missing. Values represent mean ± standard deviation, number of subjects (%), or median (Q1–Q3). There were no statistically significant differences between the periodontitis and the controls.

### Dental Health

**FIGURE 1:**
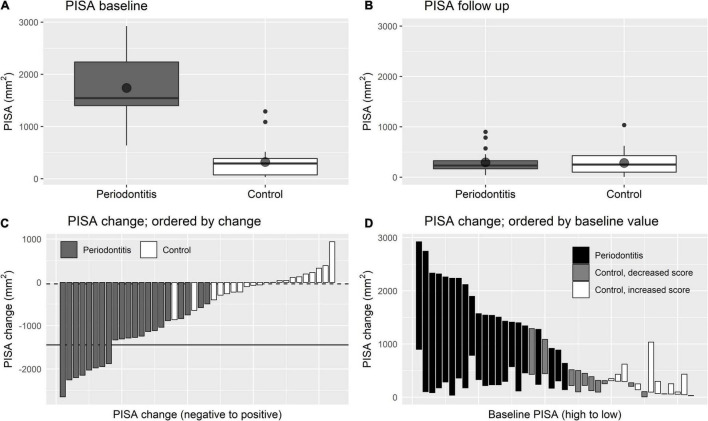
Changes in PISA score. **(A,B)** Are boxplots showing the distribution of PISA score at baseline and at follow up, respectively. Point shapes indicate sample means. **(C,D)** Are waterfall plots showing the change of PISA score while ordered by the extent of PISA change and ordered by baseline PISA score, respectively. In **(C)** the solid line indicates mean PISA change for periodontitis patients and the dashed line indicates mean change for controls.

Figure 1 and Table 2 show baseline values, 1-year follow-up values and changes in dental health. The inflammatory burden of the periodontitis patients improved significantly after periodontal treatment: 1 year after completing the periodontal treatment, the mean decrease of the PISA score was −1444.8 mm^2^ ± 612.4 (*p* < 0.001). In the control group, mean decrease of the PISA score was −35.6 mm^2^ (*p* = 0.670). Figure 1C visualizes an association of baseline PISA score with PISA change.

**TABLE 2:** Changes in dental health.

	Baseline	Follow-up	Change	*p*-value
**All**				
PISA score	1030.1 ± 868.0	290.0 ± 226.0	−740.2 ± 872.2	<0.001
Teeth	28.0 (26.0–28.0)	27.0 (25.0–28.0)	0.0 (−0.3 to 0.0)	0.005
Plaque score	40.6 ± 28.1	15.1 ± 9.9	−25.5 ± 30.1	<0.001
Bleeding score	43.8 ± 31.1	15.1 ± 9.8	−28.7 ± 33.2	<0.001
**Periodontitis**				
PISA score	1740.7 ± 612.5	295.9 ± 221.6	−1444.8 ± 612.4	<0.001
Teeth	28.0 (24.0–28.5)	26.0 (22.0–28.0)	0.0 (−2.5 to 0.0)	0.017
Plaque score	60.6 ± 19.4	12.8 ± 7.2	−47.8 ± 19.1	<0.001
Bleeding score	67.6 ± 18.4	13.2 ± 9.0	−54.4 ± 19.9	<0.001
**Control**				
PISA score	319.6 ± 330.3	284.0 ± 235.7	−35.6 ± 376.6	0.670
Teeth	28.0 (26.0–28.0)	28.0 (26.0–28.0)	0.0 (0.0–0.0)	0.102
Plaque score	20.7 ± 20.0	17.4 ± 11.7	−3.3 ± 21.2	0.485
Bleeding score	20.0 ± 21.2	17.1 ± 10.3	−2.9 ± 21.6	0.544

*Values represent mean ± standard deviation, or median (Q1–Q3). Group differences were tested by a paired t-test or Wilcoxon signed rank test.*

The mean decrease of the plaque score was −47.8% ± 19.1 and the mean decrease of the bleeding score was −54.4% ± 19.9. Due to selective tooth extraction, as part of the periodontal treatment, the number of teeth also significantly decreased (*p* = 0.017). The dental health of the control group did not significantly change during the follow-up period.

### Cardiovascular Health

**FIGURE 2:**
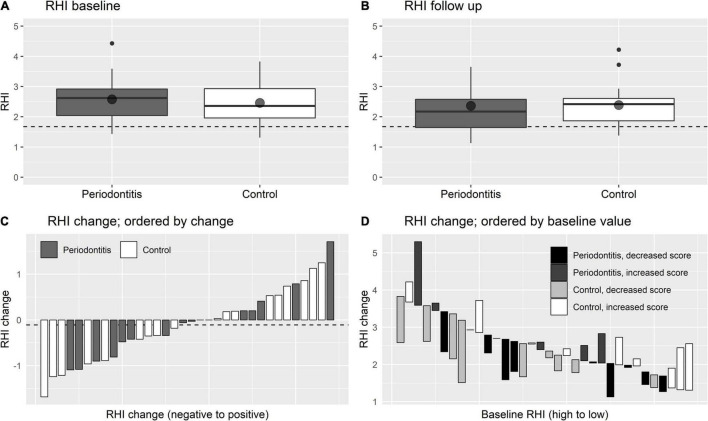
Changes in endothelial function (RHI). **(A,B)** Are boxplots showing the distribution of RHI at baseline and at follow up, respectively Point shapes indicate sample means. The dashed line presents RHI cut-off score of 1.67 that correlates to endothelial dysfunction. **(C,D)** Are waterfall plots showing the change of RHI while ordered by the extent of RHI change and ordered by baseline RHI, respectively. In **(C)** the dotted line indicates mean RHI change for periodontitis patients.

Data on cardiovascular conditions at baseline and after the follow-up period of the periodontitis and controls are shown in Table 3. Endothelial function, the primary outcome, expressed as RHI, did not show a significant improvement after treatment of the periodontitis patients (RHI −0.1 ± 0.8, *p* = 0.524). Figures 2A,B illustrates the minor changes in endothelial function, expressed by the RHI, observed from baseline to 1 year after completing periodontal therapy. In Figures 2C,D we present waterfall plots to demonstrate the change of the endothelial function ordered by the extent of the RHI change and ordered by the RHI at baseline. Due to technical problems with the EndoPAT™, the RHI of 8 subjects (6 periodontitis patients and 2 control subjects) was unknown. Similarly, all other secondary cardiovascular outcome measurements, hsCRP, total cholesterol, HDL cholesterol, triglycerides, HbA1c, and systolic blood pressure did not significantly improve after periodontal therapy.

**TABLE 3:** Changes in cardiovascular conditions.

	Baseline	Follow-up	Change	*p*-value
**All**				
Endothelial function (RHI)	2.5 ± 0.7	2.4 ± 0.9	−0.1 ± 0.8	0.424
hsCRP	1.1 (0.6–2.3)	0.75 (0.0–1.9)	−0.3 (−0.6 to 0.3)	0.123
Total cholesterol	5.4 ± 0.8	5.3 ± 0.8	−0.1 ± 0.7	0.350
HDL cholesterol	1.7 ± 0.5	1.7 ± 0.4	0.0 ± 0.2	0.705
LDL cholesterol	3.3 ± 0.9	3.2 ± 0.8	−0.1 ± 0.6	0.173
Triglycerides	1.0 (0.7–1.2)	0.9 (0.7–1.1)	0.0 (−0.2 to 0.1)	0.634
HbA1c	36.1 ± 2.7	36.3 ± 2.6	0.3 ± 1.3	0.195
Systolic BP	126.3 ± 15.2	123.2 ± 11.3	−3.1 ± 13.4	0.113
**Periodontitis**				
Endothelial function (RHI)	2.5 ± 0.6	2.4 ± 1.0	−0.1 ± 0.8	0.524
hsCRP	1.2 (0.6–2.8)	1.0 (0.0–2.5)	−0.3 (−0.7 to 0.5)	0.339
Total cholesterol	5.2 ± 0.8	5.1 ± 0.7	−0.1 ± 0.4	0.185
HDL cholesterol	1.6 ± 0.5	1.6 ± 0.4	−0.0 ± 0.2	0.787
LDL cholesterol	3.1 ± 0.8	3.0 ± 0.7	−0.2 ± 0.4	0.101
Triglycerides	1.0 (0.6–1.4)	0.9 (0.8–1.4)	0.0 (−0.2 to 0.4)	0.490
HbA1c	36.9 ± 2.6	36.8 ± 2.5	−0.0 ± 1.2	0.858
Systolic BP	129.3 ± 16.6	125.4 ± 11.0	−3.9 ± 13.8	0.208
**Control**				
Endothelial function (RHI)	2.5 ± 0.8	2.4 ± 0.7	−0.1 ± 0.8	0.623
hsCRP	0.9 (0.6–1.7)	0.6 (0.0–1.4)	−0.2 (−0.4 to 0.1)	0.169
Total cholesterol	5.7 ± 0.8	5.6 ± 0.9	−0.1 ± 0.9	0.693
HDL cholesterol	1.7 ± 0.4	1.8 ± 0.4	0.0 ± 0.2	0.438
LDL cholesterol	3.5 ± 0.9	3.4 ± 0.9	−0.1 ± 0.7	0.578
Triglycerides	0.9 (0.7–1.2)	0.8 (0.7–1.1)	−0.1 (−0.2 to 0.0)	0.099
HbA1c	35.3 ± 2.6	35.9 ± 2.6	0.6 ± 1.3	0.062
Systolic BP	123.3 ± 13.4	121.1 ± 11.4	−2.3 ± 11.1	0.357

*RHI missing at follow-up: 8 (6 periodontitis and 2 control). Values represent mean ± standard deviation, or median (Q1–Q3). Group differences were tested by a paired t-test or Wilcoxon signed rank test.*

After the 1-year follow-up period of the controls, none of the cardiovascular variables significantly changed.

### Association Between Periodontal Improvement and Cardiovascular Health

**FIGURE 3:**
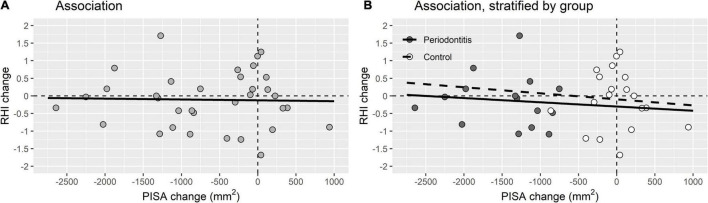
Association between periodontal improvement and endothelial function. **(A)** Association between PISA change and RHI change for the total study population (*R*^2^ linear = 6.549E-4). **(B)** Association between PISA change and RHI change stratified by periodontitis patients (*R*^2^ linear = 0.008) and control group (*R*^2^ linear = 0.006) **(B)**.

Table 4 emphasizes that there were no significant differences in change of the cardiovascular variables after treatment of the periodontitis patients compared to the control group. Figure 3 illustrates that there was no significant association between the decrease of the PISA score, periodontal improvement, and the endothelial function, expressed in RHI.

**TABLE 4:** Differences in cardiovascular change between periodontitis patients and controls.

	Change periodontitis	Change control	Difference	*p*-value
**All**				
Endothelial function (RHI)	−0.1 ± 0.8	−0.1 ± 0.8	−0.1 (NA)	0.960
hsCRP	−0.3 (−0.7 to 0.5)	−0.2 (−0.4 to 0.1)	−0.1 (NA)	0.880
Total cholesterol	−0.1 ± 0.4	−0.1 ± 0.9	−0.0 (−0.5 to 0.4)	0.839
HDL cholesterol	−0.0 ± 0.2	0.0 ± 0.2	−0.1 (−0.2 to 0.1)	0.452
LDL cholesterol	−0.2 ± 0.4	−0.1 ± 0.7	−0.1 (−0.4 to 0.3)	0.689
Triglycerides	0.0 (−0.2 to 0.4)	−0.1 (−0.2 to 0.0)	−0.1 (NA)	0.232
HbA1c	−0.0 ± 1.2	0.6 ± 1.3	−0.6 (−1.4 to 0.2)	0.121
Systolic BP	−3.9 ± 13.8	−2.3 ± 11.1	−1.6 (−9.4 to 6.2)	0.677

*Values represent mean ± standard deviation, or median (Q1–Q3). Group differences were tested by an independent t-test or Mann-Whitney U-test.*

## Discussion

The current clinical intervention follow-up study aimed to investigate the effect of periodontal treatment on endothelial function, in otherwise healthy adults. The clinical results showed that significant decrease of the inflammatory burden after periodontal treatment did not improve the endothelial function, as measured by the RHI with the EndoPAT™, or other cardiovascular parameters after 1-year follow-up.

Positive effects of periodontal treatment on endothelial function and other cardiovascular parameters that were demonstrated in previous intervention studies, focused more on patients already suffering from CVD. Correspondingly, levels of hsCRP of the included untreated periodontitis patients were somewhat lower than previously studies reported (27, 28). In this study we focused on otherwise healthy adults, without a history of CVD. Only 5 participants suffered from endothelial dysfunction at baseline. The mean baseline endothelial function, expressed in RHI, in our study population was higher than the RHI cut-off score of 1.67 that correlates to endothelial dysfunction. Probably, because the RHI at baseline was well within normal limits, a positive effect of periodontal treatment could not be expected. Besides, we used the EndoPAT™ device for measuring endothelial function. This device might not be sensitive enough to detect small differences in endothelial function.

Some systematic reviews support the positive effects of periodontal treatment on CVD risk parameters, especially 6 months after the treatment. This effect extinguished after a follow-up time of 12 months (29). We have chosen consciously for a follow-up period of 1 year, because intervention studies with a 1-year follow-up time are scarce. Furthermore, an even more extended follow-up time, with cardiovascular events as hard endpoint, is needed to confirm or reject the causal relation between periodontitis and CVD. However, these kind of studies are challenging, due to methodological, financial and most important, ethical considerations (30).

Despite numerous publications investigating the association between periodontitis and CVD, there is still no consensus whether periodontitis plays a pathophysiological role in CVD (8). Ever since the publication of the first studies indicating an association, this topic received substantial professional and public interest. Consequently, this subject generated debates between researchers, caused wide-scale media coverage and prompted involved professional organizations to issue official statements. It remains essential to understand the quality of the underlying literature to be able to perform a critical appraisal. Regrettably, we must be aware of poor reporting and misinforming, concerning the clinical trials evaluating the effect of periodontal treatment on CVD (31).

A limitation of this study is the relatively small study group. Although the effect of periodontal treatment on endothelial function in periodontitis patients has been suggested in the literature, this has rarely been investigated in exclusively, otherwise healthy periodontitis patients. We are the first study that investigated asymptomatic healthy periodontitis patients from the dental practice, using the endothelial function assessment by the EndoPAT. We focused on this specific patient population, to investigate whether the apparently otherwise healthy periodontitis patients in the dental practice are possibly at risk for a CVD event in the future and whether periodontal treatment can reduce such risk for a CVD event. Due to this explorative nature of the study, a proper power-analysis was not applicable. In retrospect, enlargement of the study population would have strengthened the current study. Besides, we included patients who visited a specialized dental clinic for periodontology. It must be taken into account that these patients may not fully representative of the general population.

With the current pilot study, we have attempted to add further knowledge to the once-wide gap between dentistry and general medicine, aimed to identify patients at risk for CVD in an earlier stage. In conclusion, periodontitis and CVD are complex inflammatory diseases with shared modifiable and non-modifiable risk factors. Periodontal treatment as primary or secondary prevention of CVD could be focused on direct control of periodontitis and changing modifiable risk factors of both. In this study, we did not find an improvement of endothelial function or other cardiovascular parameters after highly effective periodontal treatment including non-surgical and surgical therapy. Future studies are needed to be a larger size and could focus on periodontitis patients with co-morbidities, whether periodontal treatment has secondary preventive effect on endothelial function and other CVD parameters.

## Data Availability Statement

The raw data supporting the conclusions of this article will be made available by the authors, without undue reservation.

## Ethics Statement

This study was approved by the Medical Ethics Committee, Isala Academy, Zwolle, the Netherlands (NL43083.075.13). The patients/participants provided their written informed consent to participate in this study.

## Author Contributions

HD, EV, AH, JL, and BL contributed to conception and design of the study. EV and HD included the patients and arranged the clinical measurements. HD organized the database and wrote the first draft of the manuscript. HD and ME performed the statistical analysis. HD, ME, AH, JL, and BL wrote sections of the manuscript. All authors contributed to manuscript revision, read, and approved the submitted version.

## Conflict of Interest

The authors declare that the research was conducted in the absence of any commercial or financial relationships that could be construed as a potential conflict of interest.

## Publisher’s Note

All claims expressed in this article are solely those of the authors and do not necessarily represent those of their affiliated organizations, or those of the publisher, the editors and the reviewers. Any product that may be evaluated in this article, or claim that may be made by its manufacturer, is not guaranteed or endorsed by the publisher.
